# The contribution of multiple long-term conditions to widening inequalities in disability-free life expectancy over two decades: Longitudinal analysis of two cohorts using the Cognitive Function and Ageing Studies

**DOI:** 10.1016/j.eclinm.2021.101041

**Published:** 2021-07-31

**Authors:** Holly Q Bennett, Andrew Kingston, Ilianna Lourida, Louise Robinson, Lynne Corner, Carol EG Brayne, Fiona E Matthews, Carol Jagger

**Affiliations:** a Population Health Sciences Institute, Faculty of Medical Sciences, Biomedical Research Building, Campus for Ageing and Vitality, Newcastle University, Newcastle upon Tyne, NE4 5PL, UK; b Cambridge Institute of Public Health, Forvie site, University of Cambridge School of Clinical Medicine, Cambridge Biomedical campus, Cambridge CB2 0SR, UK; c Population Health Sciences Institute, Faculty of Medical Sciences, Newcastle University, Edwardson Building, Campus for Ageing and Vitality, Newcastle upon Tyne NE4 5PL, UK

**Keywords:** Life expectancy, Health expectancy, Disability, Multimorbidity, Socioeconomic status

## Abstract

**Background:**

: Disability-free life expectancy (DFLE) inequalities by socioeconomic deprivation are widening, alongside rising prevalence of multiple long-term conditions (MLTCs). We use longitudinal data to assess whether MLTCs contribute to the widening DFLE inequalities by socioeconomic deprivation.

**Methods:**

: The Cognitive Function and Ageing Studies (CFAS I and II) are large population-based studies of those ≥65 years, conducted in three areas in England. Baseline occurred in 1991 (CFAS I, *n*=7635) and 2011 (CFAS II, *n*=7762) with two-year follow-up. We defined disability as difficulty in activities of daily living, MLTCs as the presence of at least two of nine health conditions, and socioeconomic deprivation by area-level deprivation tertiles. DFLE and transitions between disability states and death were estimated from multistate models.

**Findings:**

: For people with MLTCs, inequalities in DFLE at age 65 between the most and least affluent widened to around 2.5 years (men:2.4 years, 95% confidence interval (95%CI) 0.4–4.4; women:2.6 years, 95%CI 0.7–4.5) by 2011. Incident disability reduced for the most affluent women (Relative Risk Ratio (RRR):0.6, 95%CI 0.4–0.9), and mortality with disability reduced for least affluent men (RRR:0.6, 95%CI 0.5–0.8). MLTCs prevalence increased only for least affluent men (1991: 58.8%, 2011: 66.9%) and women (1991: 60.9%, 2011: 69.1%). However, DFLE inequalities were as large in people without MLTCs (men:2.4 years, 95%CI 0.3–4.5; women:3.1 years, 95% CI 0.8–5.4).

**Interpretation:**

: Widening DFLE inequalities were not solely due to MLTCs. Reduced disability incidence with MLTCs is possible but was only achieved in the most affluent.

**Research in context**

**Evidence before this study**

After searching multiple databases, one cross-sectional and six longitudinal panel studies of moderate quality reported disability-free life expectancy (DFLE) comparisons for multiple long-term conditions (MLTCs) including diabetes, cardiovascular diseases, depression and sensory impairment. Evidence was limited but suggests that LE and DFLE were generally reduced for those with MLTCs, though the impact of MLTCs may not be additive but dependent on the combination of conditions studied. We identified no studies examining the impact of MLTCs on DFLE by socioeconomic groups.

**Added value of this study**

Our study is the first to investigate the contribution of MLTCs to widening inequalities in DFLE by socioeconomic deprivation. We found that the least affluent older people with MLTCs spent a greater proportion of remaining life with disability in 2011 than 1991, providing some evidence that MLTCs may have become more disabling in this group. However, although MLTCs may be contributing to the widening LE and DFLE inequalities seen in the UK, it is not the full story, as DFLE inequalities for those without MLTCs were of a similar magnitude to those with MLTCs.

**Implications of all the available evidence**

This study suggests that a reduction in disability incidence or a delay in the onset of disability could occur, even in the presence of MLTCs. However, to date, this has only been achieved for the most affluent older women. These results, and the greater impact of Covid-19 on disadvantaged communities, render it unlikely that the Ageing Grand Challenge, of increasing healthy life expectancy by five years before 2035 while also reducing inequalities, will be met.

Alt-text: Unlabelled box

## 1 Introduction

Inequalities in life expectancy (LE) at birth in the UK have been widening for some time [1] and currently stand at ten years for men and eight years for women [2], with important sex differences. Before the recent COVID-19 pandemic, all men had seen increases in LE at birth, but gains were greatest in the least deprived. In contrast, for the most deprived women, LE fell by 93.5 days between 2013–2015 and 2016–2018. Since the pandemic, LE at birth has fallen further, for women by 0.9 years, and for men by 1.2 years [3] .Healthy life expectancy at birth, which combines health and mortality in one measure, shows even greater inequalities across socioeconomic groups, [2] being 18.3 years for males, and 18.8 years for females [2]. Previous work with the Cognitive Function and Ageing Studies (CFAS) has shown that, between 1991 and 2011, inequalities in disability-free life expectancy (DFLE) at age 65 increased by 1.7 years for men and 2.4 years for women. This resulted from improvements in the incidence of and recovery from disability in the most affluent men and women [4].

Widening inequalities in life expectancy at birth are due to higher death rates from respiratory disease, ischaemic heart disease and dementias in the least affluent [5]. Additionally, there is evidence that inequalities in life expectancy at age 65 are a result of delayed onset of multiple long-term conditions (MLTCs) of two years for men and three for women, together with longer survival with MLTCs for men of one year [6]. Recent evidence from Canada suggests that the prevalence of MLTCs has increased by 12% over the last decade, even after allowance for the increased ageing of the population. Earlier detection, in particular with hypertension, diabetes and mild cognitive impairment, may however also be a contributing factor [7].

There has been less research on the impact of MLTCs on DFLE. The majority of studies investigate combinations of two conditions only, or the presence of comorbidity with a specific condition. In these studies, the presence of other conditions tended to reduce LE and DFLE compared to having the health condition alone [8,9]. Only one study to date has reported the effect of cumulative MLTCs on DFLE, [10] and another has investigated the effect of combinations of conditions on DFLE in different racial groups [11]. However, no-one has researched whether MLTCs contribute to trends in socio-economic inequalities in DFLE.

We aim to address these shortcomings and determine whether widening DFLE inequalities between the most and least affluent older men and women in the UK is because of a greater increase in the prevalence of MLTCs in the least affluent or because MLTCs have become less disabling for the most affluent.

## 2 Methods

### 2.1 Data

**Fig. 1:**
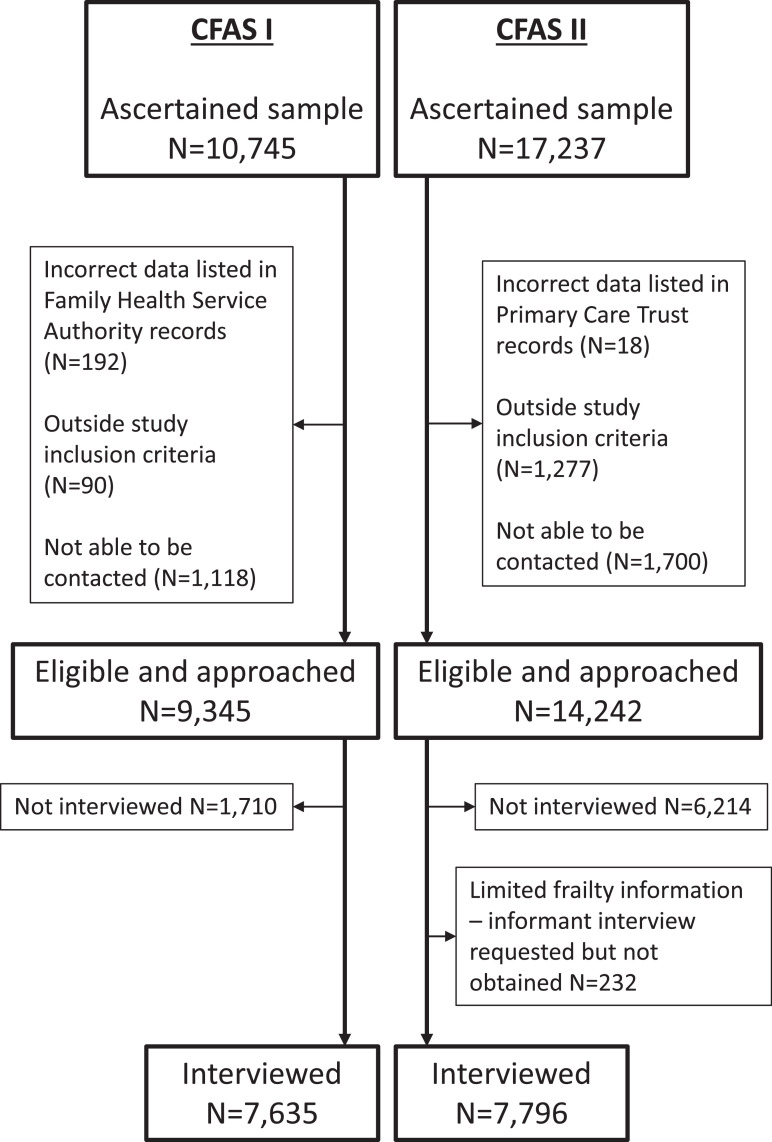
Flow chart of individuals through the Cognitive Function and Ageing Studies (CFAS I and CFAS II) from ascertainment to inclusion in the study.

The Cognitive Function and Ageing Studies (CFAS I and CFAS II) are two large population based longitudinal studies of those aged 65 and over conducted two decades apart. Both were based in the same three areas of England – Cambridgeshire, Nottingham and Newcastle upon Tyne. For each study a random sample from the Family Health Service Authority lists were approached through letter jointly signed by their general practitioner and local principal investigator. Sampling was stratified into age groups 65–74 and 75 and over and with an identical sampling strategy in CFAS I and CFAS II. If the participant agreed, they were asked to sign a consent form and a mutually convenient time was agreed for the interviewer to visit them in their home, where responses to the questionnaire were recorded digitally. Fig. 1 shows the numbers of individuals from ascertainment to participation in CFAS I and CFAS II, and has previously been published in greater detail [12]. Baseline interviews began in 1991 for CFAS I, and 2008 for CFAS II, with follow up interviews conducted two years later. Anyone who had participated at baseline and were still alive were re-approached and anyone who did not refuse, move or get lost to follow-up were re-interviewed. A subsample of participants nominated a friend or family member for an informant interview, which we used to substitute missing data in the participant interview. The Office for National Statistics (ONS) supplied date of death routinely and was available up to 4.5 years after baseline interviews. For further detail please see the study protocol on the CFAS website [13,14].

### 2.2 Measures

The disability measure was based on the modified Townsend activities of daily living scale [15,16]. Being unable to leave their house or requiring help with washing all over, preparing and cooking a hot meal, putting on shoes and socks, heavy housework or shopping and carrying heavy bags would categorise an individual as having disability. If no help was required and they were ambulant outside their house then they would be disability-free. The Townsend deprivation index [17], a measure of material index based on locality data applied to individuals based on their postcode, was used as a measure of socioeconomic deprivation and split into tertiles for each study. The multiple long-term conditions (MLTCs) measure comprised having two or more of: arthritis, coronary heart disease (angina or heart attack), diabetes, peripheral vascular disease, respiratory difficulties (asthma or bronchitis), stroke, hearing difficulties, visual impairment, or cognitive impairment. Cognitive impairment was determined from a Mini Mental State Examination (MMSE) [18] score of 25 or less. Hearing difficulties and visual impairment were a combination of self-report and interviewer rating. The interviewer was asked whether poor eyesight or hearing problems interfered with the interview and could give one of the following options: no, to some extent, to a marked extent or blind/deaf. All other health conditions were self-report of a doctor diagnosis. If a participant was missing data on any of the health conditions, they were considered to have MLTCs if the percentage of the health conditions present was ≥22.2% (equivalent to two or more out of nine health conditions). For example, if information was only present on 6 of the health conditions for a participant, this would not be included as having missing MLTCs as they do not have a record of health conditions, instead if they had 2 out of 6 of the health conditions recorded (33.3% > 22.2%), they would be recorded as having MLTCs.

### 2.3 Statistical methods

**Fig. 2:**
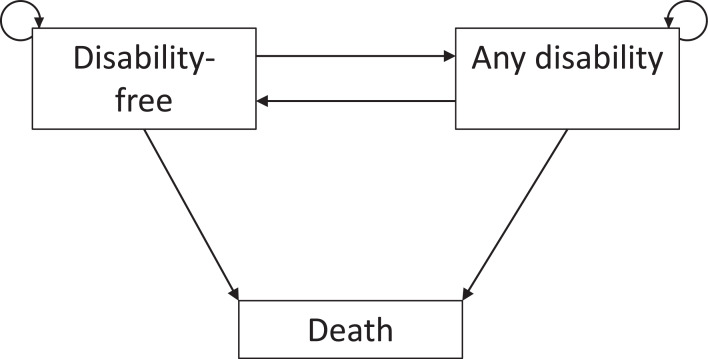
Transitions in the multistate models used to estimate disability-free life expectancy, years with disability and transition probabilities.

The prevalence of MLTCs was estimated at baseline and two year follow up. Decline and recovery transitions between disability-free, disability and death were estimated through a three-state multistate model, and then combined to estimate life and health expectancies in Interpolated Markov Chain software (IMaCh [19] version 0.99r19). Fig. 2 describes the transitions being estimated by the multistate model, including remaining in one of the alive states, transitioning to disability (incident disability), recovery from disability to disability-free and transitioning from either disability-free or with disability to death. In IMaCh, time is modelled discretely in steps through multinomial logistic regression, with the steps starting at two years and wherever possible gradually being decreased to one month to become closer to continuous time, for more detail see appendix. Models for LE and DFLE were stratified by study and sex, as LE and DFLE differ greatly between studies and sexes, and adjusted for deprivation and MLTCs. Steps could be decreased to one month. These models were also used to estimate state-based life and health expectancies for those initially free of disability. To estimate the relative probability of transitioning between each state in CFAS II compared to CFAS I, models were stratified by sex, deprivation and MLTCs, and adjusted for study. The stratified groups did not have as large numbers and therefore steps could only be reduced to six months. Inverse probability weights for longitudinal attrition including age, sex, study centre, deprivation, MMSE, functional impairment, education level, social class, grouped count of health conditions, self-reported health and smoking status were multiplied by baseline weights that included age, sex, centre and deprivation to ensure population representativeness for each of the study samples. An additional weight was created for those who died, taking into account the probability of death at each year of age from national mortality data from ONS in comparison to CFAS. To validate IMaCh, we compared the estimates of LE at age 65 and 85 for men and women in CFAS I and CFAS II from IMaCh to LE estimates for England from the Office for National Statistics and found them to be close (Appendix Table 1).

### 2.4 Role of the funding source

The funding source had no role in the study design, data collection, data analysis, data interpretation, writing of the report or decision to submit the paper for publication. All authors confirm they had full access to the data in the study and accept responsibility for the decision to submit for publication.

### 2.5 Ethics

The current ethics for MRC CFAS is from Eastern MREC, reference number 05/MRE05/37 and for the mortality data Wales REC 7, reference number 14/WA/1154. The current REC reference number for CFAS II is 07/MRE05/48 from Cambridge REC 4. For further information on past ethical approvals please visit the CFAS website (www.cfas.ac.uk).

## 3 Results

### 3.1 Demographics

The average age of the 7635 participants at baseline in CFAS I (1991) was 75.6 years and 60.8% were women. Two years later, 5156 were re-interviewed, 76% of the 6816 still alive. Out of the 7762 participants at baseline in CFAS II (2011), 56.1% were women and average age was 76.4 years. There were 5288 returning participants for the two-year follow-up interview, 74% of the 7119 still alive. Total follow-up was 28930.4 years in CFAS I, on average 4.5 years and for CFAS II total follow-up was 30027.8 years, 4.7 years on average. In CFAS I 31.5% had disability at baseline (missing 1.1%) and of those who participated in two year follow-up, 37.4% had disability (missing 2.8%). At baseline in CFAS II 36.4% had disability (missing 4.0%) and at the two year follow-up 36.6% of those who participated had disability (missing 0.7%). Cut points for deprivation tertiles in CFAS I were -1.37 and 2.75, cut points for CFAS II were -2.07 and 1.11 with no missing data in CFAS I or CFAS II. The crude prevalence of MLTCs at baseline was ten percentage points higher in the least affluent men in 1991 (least affluent: 58.8%, most affluent: 47.0%), and almost 20 percentage points higher in 2011 (least affluent: 66.9%, most affluent: 47.6%), with a similar pattern in women (Table 1). The crude prevalence of MLTCs in the most affluent men and women changed little between 1991 and 2011 but increased for the least affluent (Table 1). Missing data for MLTCs was 0.1% in CFAS I and 0.4% in CFAS II. Further demographic information is provided in other CFAS papers [13,20].

**Table 1:** Number and weighted percentage of men and women in each deprivation group at baseline with multiple long term health conditions in the Cognitive Function and Ageing Studies (CFAS I and II). Percentages inverse probability weighted.

Sex	Socioeconomic deprivation	CFAS I		CFAS II	
		*n*	%	*n*	%
Men	Most affluent	506	47.0	647	47.6
	Moderately affluent	473	48.6	612	53.4
	Least affluent	573	58.8	641	66.9
Women	Most affluent	757	52.2	793	52.6
	Moderately affluent	845	55.5	816	57.4
	Least affluent	948	60.9	802	69.1

### 3.2 Life expectancies and disability-free life expectancies at age 65

**Fig. 3:**
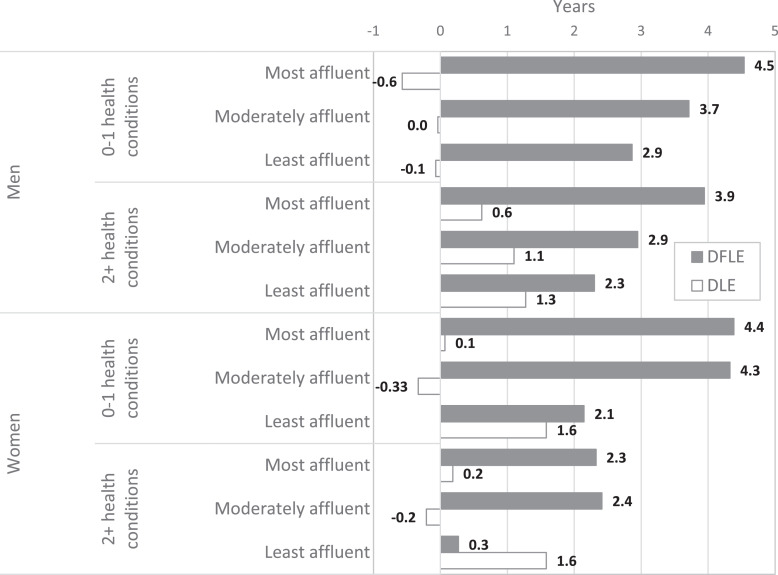
Gain (+) and loss (-) in disability-free life expectancy (DFLE) and years with disability (DLE) between the Cognitive Function and Ageing Studies (CFAS I and CFAS II).

In the presence of MLTCs, increases in disability-free years between 1991 and 2011 were greater for the most affluent men whereas increases in years with disability were greater for the least affluent men (Fig. 3). Therefore, by 2011, although the percentage of life spent with disability was still greater for men with MLTCs, it was no longer similar across deprivation groups, with the least affluent men spending almost a third of their remaining life with disability (Table 2). In 1991, MLTCs were associated with increased risk of incident disability and increased risk of death with disability in men (Table 3). By 2011 MLTCs were again associated with increased risk of incident disability and men in the less affluent group were less likely to recover from disability (Table 3).

**Table 2:** Life expectancy (LE), disability-free life expectancy (DFLE) and life expectancy with disability (DLE) at age 65 by sex, socio-economic deprivation and with 0-1 health conditions or 2+ health conditions in the Cognitive Function and Ageing Studies (CFAS I and II). Difference between most and least affluent in each category.

Sex	Socioeconomic deprivation		CFAS I				CFAS II			
			0–1 health conditions		2+ health conditions		0–1 health conditions		2+ health conditions	
			Years	%	Years	%	Years	%	Years	%
Men	Most affluent	LE	16.6 (15.4–17.7)		12.5 (11.5–13.4)		20.6 (19.2–21.9)		17.0 (15.8–18.3)	
		DFLE	12.8 (11.6–14.1)	77.3	8.8 (7.7–9.9)	70.7	17.4 (15.9–18.8)	84.5	12.7 (11.3–14.1)	74.9
		DLE	3.8 (3.0–4.5)	22.7	3.7 (3.0–4.3)	29.3	3.2 (2.4–4.0)	15.5	4.3 (3.5–5.0)	25.1
	Moderately affluent	LE	16.5 (15.2–17.8)		12.4 (11.3–13.5)		20.2 (18.8–21.6)		16.4 (15.3–17.6)	
		DFLE	12.9 (11.5–14.3)	78.2	8.9 (7.6–10.1)	71.4	16.6 (15.1–18.2)	82.3	11.8 (10.4–13.2)	71.8
		DLE	3.6 (2.8–4.4)	21.8	3.5 (2.9–4.2)	28.6	3.6 (2.7–4.4)	17.7	4.6 (3.9–5.4)	28.2
	Least affluent	LE	15.9 (14.5–17.2)		11.7 (10.7–12.8)		18.7 (17.2–20.1)		15.3 (14.2–16.4)	
		DFLE	12.1 (10.6–13.5)	76.0	8.1 (6.9–9.3)	68.9	14.9 (13.4–16.5)	80.0	10.4 (9.0–11.7)	67.8
		DLE	3.8 (2.9–4.7)	24.0	3.6 (3.0–4.3)	31.1	3.7 (2.8–4.7)	20.0	4.9 (4.1–5.8)	32.2
	*Difference most-least affluent*	*LE*	*0.7 (-1.1–2.5)*		*0.7 (-0.7–2.2)*		*1.9 (-0.1–3.9)*		*1.7 (0.1–3.4)*	
		*DFLE*	*0.8 (-1.2–2.7)*		*0.7 (-0.9–2.4)*		*2.4 (0.3–4.5)*		*2.4 (0.4–4.3)*	
		*DLE*	*−0.05 (−1.2–1.1)*		*0.01 (−0.9–0.9)*		*−0.5 (−1.8–0.7)*		*−0.6 (−1.8–0.5)*	
Women	Most affluent	LE	19.9 (18.8–21.1)		17.3 (16.4–18.2)		24.4 (22.8–26.0)		19.8 (18.6–21.0)	
		DFLE	12.0 (10.9–13.1)	60.2	8.4 (7.4–9.4)	48.6	16.4 (14.7–18.1)	67.2	10.7 (9.3–12.2)	54.2
		DLE	7.9 (7.0–8.9)	39.8	8.9 (8.0–9.7)	51.4	8.0 (6.7–9.3)	32.8	9.1 (7.9–10.3)	45.9
	Moderately affluent	LE	19.1 (17.8–20.5)		16.8 (15.6–18.0)		23.1 (21.5–24.7)		19.0 (17.6–20.3)	
		DFLE	11.4 (10.1–12.6)	59.5	8.0 (6.9–9.1)	47.5	15.7 (14.0–17.3)	67.9	10.4 (8.9–11.8)	54.7
		DLE	7.7 (6.6–8.9)	40.5	8.8 (7.7–9.9)	52.5	7.4 (6.2–8.7)	32.1	8.6 (7.4–9.8)	45.3
	Least affluent	LE	18.2 (16.8–19.6)		15.9 (14.7–17.1)		21.9 (20.2 – 23.6)		17.7 (16.6–18.8)	
		DFLE	11.2 (9.9–12.4)	61.4	7.9 (6.8–8.9)	49.5	13.3 (11.7–14.9)	60.7	8.1 (6.9–9.4)	45.9
		DLE	7.0 (5.9–8.1)	38.6	8.0 (7.0–9.0)	50.5	8.6 (7.2–10.0)	39.3	9.6 (8.4–10.8)	54.2
	*Difference most-least affluent*	*LE*	*1.8 (−0.1–3.6)*		*1.4 (−0.1–2.9)*		*2.5 (0.2–4.8)*		*2.1 (0.4–3.7)*	
		*DFLE*	*0.8 (−0.8–2.5)*		*0.5 (−0.9–1.9)*		*3.1 (0.8–5.4)*		*2.6 (0.7–4.5)*	
		*DLE*	*0.9 (−0.5–2.4)*		*0.9 (−0.5–2.2)*		*−0.6 (−2.5–1.3)*		*−0.5 (−2.2–1.2)*

**Table 3:** Relative risk ratio (RRR) and 95% confidence intervals (CI) of transitioning between disability states and death for men from different socioeconomic deprivation groups and with and without multiple long-term conditions (MLTCs) adjusted as covariates, and stratified by study (Cognitive Function and Ageing Studies CFAS I and CFAS II).

		CFAS I		CFAS II	
Transition	Covariate	RRR	95% CI	RRR	95% CI
No disability -> Disability	Most affluent	Ref.		Ref.	
	Moderately affluent	1.1	(0.9–1.5)	1.1	(0.8–1.5)
	Least affluent	1.2	(0.9–1.6)	1.2	(0.9–1.6)
	0-1 health conditions	Ref.		Ref.	
	2+ health conditions	1.8	(1.4–2.3)	2.2	(1.6–3.0)
No disability -> Death	Most affluent	Ref.		Ref.	
	Moderately affluent	1.0	(0.7–1.5)	0.9	(0.5–1.6)
	Least affluent	1.0	(0.6–1.4)	1.3	(0.7–2.1)
	0-1 health conditions	Ref.		Ref.	
	2+ health conditions	1.3	(0.9–1.9)	1.1	(0.7–1.7)
Disability -> No disability	Most affluent	Ref.		Ref.	
	Moderately affluent	1.5	(0.9–2.4)	0.8	(0.5–1.3)
	Least affluent	1.0	(0.6–1.7)	0.6	(0.4–0.9)
	0-1 health conditions	Ref.		Ref.	
	2+ health conditions	0.7	(0.4–1.3)	0.9	(0.6–1.3)
Disability -> Death	Most affluent	Ref.		Ref.	
	Moderately affluent	1.0	(0.8–1.2)	1.0	(0.8–1.3)
	Least affluent	1.1	(0.9–1.3)	1.0	(0.8–1.2)
	0-1 health conditions	Ref.		Ref.	
	2+ health conditions	1.4	(1.1–1.7)	1.1	(0.9–1.4)

The overall pattern of higher LE and DFLE in the most affluent women with MLTCs was evident in 1991 and 2011, but was more pronounced in 2011 (Table 2, Fig. 3). There is therefore some evidence that the most affluent men and the most and moderately affluent women with MLTCs spent a lower proportion of remaining life with disability in 2011 than 1991 (Table 2). For women, MLTCs were associated with increased risk of incident disability in 1991 and 2011 (Table 4). For women in 2011, having MLTCs was also associated with being less likely to recover from disability and being more likely to die with disability; being less affluent was associated with increased risk of incident disability (Table 4).

**Table 4:** Relative risk ratio (RRR) and 95% confidence intervals (CI) of transitioning between disability states and death for women from different socioeconomic deprivation groups and with and without multiple long-term conditions (MLTCs), adjusted as covariates, and stratified by study (Cognitive Function and Ageing Studies CFAS I and CFAS II).

		CFAS I		CFAS II	
Transition	Covariate	RRR	95% CI	RRR	95% CI
No disability -> Disability	Most affluent	Ref.		Ref.	
	Moderately affluent	1.0	(0.8–1.2)	1.0	(0.8–1.3)
	Least affluent	1.1	(0.9–1.3)	1.4	(1.1–1.9)
	0-1 health conditions	Ref.		Ref.	
	2+ health conditions	1.7	(1.4–2.0)	1.7	(1.3–2.1)
No disability -> Death	Most affluent	Ref.		Ref.	
	Moderately affluent	1.5	(0.8–3.2)	1.6	(0.7–3.6)
	Least affluent	1.7	(0.8–3.6)	1.4	(0.5–3.7)
	0-1 health conditions	Ref.		Ref.	
	2+ health conditions	1.0	(0.5–1.9)	1.0	(0.4–2.3)
Disability -> No disability	Most affluent	Ref.		Ref.	
	Moderately affluent	0.9	(0.6–1.3)	1.0	(0.7–1.5)
	Least affluent	1.1	(0.8–1.5)	0.9	(0.6–1.3)
	0-1 health conditions	Ref.		Ref.	
	2+ health conditions	0.7	(0.5–1.1)	0.6	(0.4–0.8)
Disability -> Death	Most affluent	Ref.		Ref.	
	Moderately affluent	1.0	(0.9–1.1)	1.0	(0.9–1.2)
	Least affluent	1.1	(1.0–1.3)	1.1	(0.9–1.3)
	0-1 health conditions	Ref.		Ref.	
	2+ health conditions	1.1	(0.9–1.3)	1.4	(1.1–1.7)

If the widening inequalities in DFLE between the most and least affluent were a result of MLTCs then we would expect smaller DFLE inequalities in older people without MLTCs. However, although there was no evidence of differences between 1991 and 2011 in inequalities in years with disability between the most and least affluent, there were increases in inequalities in years disability-free for both men and women without MLTCs. Indeed, the patterning across deprivation groups, and the magnitude of the inequalities in LE and DFLE were similar between those without and with MLTCs (Table 2, Fig. 3). Thus the widening inequalities in DFLE at age 65 over time previously found [4] were not wholly due to the increased prevalence of MLTCs since inequalities in DFLE between the most and least affluent also widened in those without MLTCs.

**Fig. 4:**
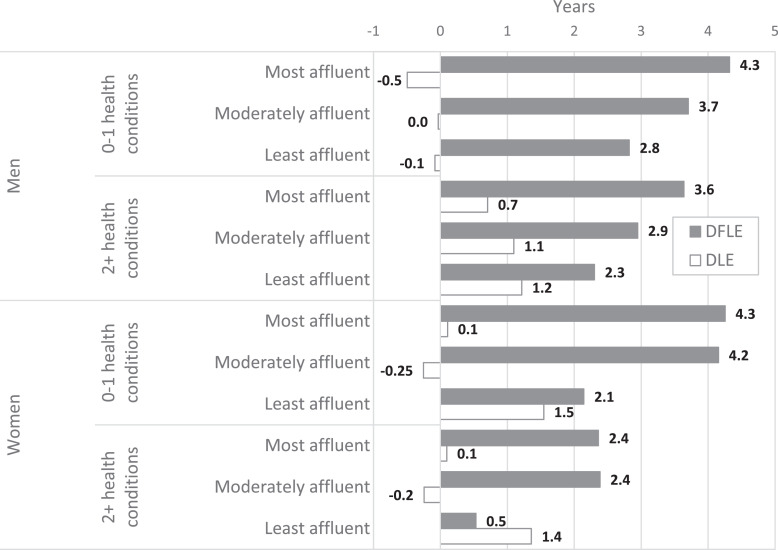
Gain (+) and loss (-) in disability-free life expectancy (DFLE) and years with disability (DLE) between the two Cognitive Function and Ageing Studies (CFAS I and CFAS II) for those disability-free at baseline.

We conducted further analyses to ascertain whether the widening DFLE inequalities in individuals without MLTCs resulted from the least affluent having either a greater prevalence of disability at baseline, or a greater incidence of MLTCs in the two-year follow-up, with therefore a subsequent faster onset of disability. Firstly, we examined the change in inequalities by social deprivation between 1991 and 2011 in those who were initially disability-free at baseline through status-based life tables. In 1991, inequalities in DFLE at age 65 between the most and least affluent who were initially disability-free and without MLTCs were small (men: 0.7 years, 95% confidence interval (CI) -1.0 to 2.4; women: 0.9 years, 95% CI -0.6 to 2.4) (Fig. 4, Table 5). By 2011, these inequalities had more than tripled (men: 2.2 years, 95% CI 0.2 to 4.2; women: 3.0 years, 95% CI 0.8 to 5.2) (Fig. 4, Table 5). Secondly, we explored whether the percentage of individuals initially free of MLTCs who acquired them by the two-year follow-up increased between 1991 and 2011, and whether these changes were different between the most and least affluent. In 2011, a slightly smaller percentage of men and women acquired MLTCs during follow-up, compared to 1991, with the least affluent men and the most affluent women showing the greatest reductions (Table 6). Thus, there is little evidence that the widening inequalities in DFLE between the most and least affluent without MLTCs were due to a greater prevalence of disability at baseline in the least affluent, or a differential incidence of MLTCs from baseline to follow-up between deprivation groups.

**Table 5:** Life expectancy (LE), disability-free life expectancy (DFLE) and life expectancy with disability (DLE) at age 65 for those disability-free at baseline by sex, socio-economic deprivation and with 0–1 health conditions or 2+ health conditions in the Cognitive Function and Ageing Studies (CFAS I and II). Difference between most and least affluent in each category.

Sex	Socioeconomic deprivation		CFAS I				CFAS II			
			0–1 health conditions		2+ health conditions		0–1 health conditions		2+ health conditions	
			Years	%	Years	%	Years	%	Years	%
Men	Most affluent	LE	16.9 (15.8–18.0)		13.0 (12.1–13.9)		20.7 (19.4–22.0)		17.3 (16.2–18.4)	
		DFLE	13.3 (12.2–14.4)	78.6	9.6 (8.6–10.5)	73.6	17.6 (16.2–19.0)	85.0	13.2 (12.0–14.4)	76.2
		DLE	3.6 (2.9–4.3)	21.4	3.4 (2.9–4.0)	26.4	3.1 (2.3–3.9)	15.0	4.1 (3.4–4.9)	23.8
	Moderately affluent	LE	16.7 (15.4–18.0)		12.8 (11.7–13.8)		20.4 (19.0–21.8)		16.8 (15.8–17.9)	
		DFLE	13.2 (12.0–14.5)	79.1	9.4 (8.4–10.5)	73.7	16.9 (15.5–18.4)	83.0	12.4 (11.2–13.6)	73.5
		DLE	3.5 (2.7–4.3)	20.9	3.4 (2.7–4.0)	26.3	3.5 (2.7–4.3)	17.0	4.5 (3.7–5.2)	26.5
	Least affluent	LE	16.2 (14.9–17.5)		12.3 (11.3–13.3)		18.9 (17.5–20.4)		15.8 (14.8–16.8)	
		DFLE	12.5 (11.2–13.8)	77.4	8.9 (7.9–9.9)	72.2	15.4 (13.9–16.8)	81.1	11.2 (10.0–12.3)	70.7
		DLE	3.7 (2.8–4.5)	22.6	3.4 (2.8–4.0)	27.8	3.6 (2.7–4.5)	18.9	4.6 (3.9–5.4)	29.3
	*Difference most-least affluent*	*LE*	*0.7 (−1.0–2.4)*		*0.7 (−0.7–2.0)*		*1.8 (−0.2–3.7)*		*1.5 (−0.01–3.0)*	
		*DFLE*	*0.7 (−1.0–2.4)*		*0.7 (−0.7–2.0)*		*2.2 (0.2–4.2)*		*2.0 (0.4–3.7)*	
		*DLE*	*−0.05 (−1.1–1.0)*		*0.0 (−0.8–0.8)*		*−0.5 (−1.6–0.7)*		*−0.5 (−1.6–0.6)*	
Women	Most affluent	LE	20.1 (18.9–21.2)		17.6 (16.7–18.5)		24.4 (22.9–26.0)		20.0 (18.8–21.3)	
		DFLE	12.4 (11.4–13.3)	61.5	9.1 (8.3–9.9)	51.6	16.6 (15.0–18.2)	67.9	11.4 (10.2–12.7)	57.0
		DLE	7.7 (6.8–8.7)	38.5	8.5 (7.7–9.3)	48.4	7.8 (6.6–9.1)	32.1	8.6 (7.5–9.7)	43.0
	Moderately affluent	LE	19.2 (17.9–20.6)		17.0 (15.8–18.2)		23.1 (21.5–24.8)		19.2 (17.8–20.6)	
		DFLE	11.7 (10.6–12.8)	61.0	8.7 (7.8–9.6)	51.0	15.9 (14.3–17.5)	68.7	11.1 (9.8–12.4)	57.7
		DLE	7.5 (6.4–8.6)	39.0	8.4 (7.3–9.4)	49.0	7.2 (6.0–8.4)	31.3	8.1 (7.0–9.2)	42.3
	Least affluent	LE	18.3 (16.9–19.7)		16.1 (14.9–17.3)		22.0 (20.3–23.7)		18.0 (16.8–19.2)	
		DFLE	11.5 (10.4–12.6)	62.8	8.5 (7.6–9.4)	52.6	13.6 (12.1–15.1)	62.0	9.0 (7.9–10.1)	50.1
		DLE	6.8 (5.8–7.8)	37.2	7.6 (6.7–8.6)	47.4	8.4 (7.0–9.7)	38.0	9.0 (7.9–10.1)	49.9
	*Difference most-least affluent*	*LE*	*1.8 (−0.03–3.6)*		*1.5 (−0.1–3.0)*		*2.5 (0.1–4.8)*		*2.0 (0.4–3.7)*	
		*DFLE*	*0.9 (−0.6–2.4)*		*0.6 (−0.6–1.8)*		*3.0 (0.8–5.2)*		*2.4 (0.8–4.1)*	
		*DLE*	*0.9 (−0.5–2.3)*		*0.9 (−0.4–2.1)*		*−0.5 (−2.3–1.3)*		*−0.4 (−1.9–1.2)*

**Table 6:** Number and weighted percentage of men and women in each deprivation group who did not have multiple long-term conditions (MLTCs) at baseline but developed them by two year follow-up in the Cognitive Function and Ageing Studies (CFAS I and II). Percentages longitudinally weighted.

		CFAS I		CFAS II	
		n	%	n	%
Men	Most affluent	84	19.1	93	16.9
	Moderately affluent	95	24.7	88	21.3
	Least affluent	83	28.2	53	22.9
Women	Most affluent	124	23.2	98	17.4
	Moderately affluent	141	27.8	106	22.9
	Least affluent	142	33.3	74	30.4

Finally, we investigated which underlying transitions (incidence of, and recovery from, disability, and mortality) were contributing to the increase in inequalities in DFLE over time in those with and without MLTCs. For men with MLTCs, the wider DFLE inequalities by 2011 stemmed from reductions in the risk of death from a disability-free state in the most affluent men, and reductions in the risk of death with disability for the least affluent men (Table 7), resulting in the most affluent men living longer disability-free but the least affluent living longer with disability. The greater inequalities in women with MLTCs in 2011 compared to 1991 stemmed from the most and moderately affluent women having a lower incidence of disability in 2011 than 1991 (Table 7). For those without MLTCs, the most affluent saw both reductions in the incidence of disability between 1991 and 2011 (men and women), and in the risk of death from disability-free (men), or from disability (women) (Table 7). In contrast, the least affluent men and women without MLTCs saw little change in any transitions, with only a decrease in incident disability for men. Women in the middle deprivation group without MLTCs had evidence of improvements in the risk of recovery from disability, explaining the smaller increases in DLE between 1991 and 2011 compared to the most affluent (Table 7).

**Table 7:** Relative Risk Ratio (RRR) of transitioning between disability states in the second Cognitive Function and Ageing Study (CFAS II) compared to CFAS I, by socioeconomic deprivation and number of multiple long-term conditions (MLTC), 95% confidence interval (CI) in parenthesis. Models stratified by sex, SES and MLTCs with study as a covariate. Step m = 6 months (see methods).

			RRR (95% CI)			
Sex	Multiple Long Term Conditions	Socioeconomic deprivation	No disability to disability	No disability to death	Disability to no disability	Disability to death
Men	0-1 health conditions	Most affluent	0.6 (0.4–1.0)	0.5 (0.3–0.8)	2.1 (0.7–6.4)	1.3 (0.8–2.1)
		Moderately affluent	0.7 (0.4–1.1)	0.6 (0.4–1.1)	1.0 (0.4–2.5)	0.9 (0.5–1.6)
		Least affluent	0.5 (0.3–0.9)	0.7 (0.4–1.4)	0.6 (0.2–2.1)	0.7 (0.4–1.3)
	2+ health conditions	Most affluent	1.0 (0.7–1.5)	0.5 (0.3–0.8)	1.7 (0.8–3.4)	0.9 (0.7–1.2)
		Moderately affluent	0.7 (0.4–1.0)	0.4 (0.2–0.8)	1.0 (0.5–2.0)	0.8 (0.6–1.0)
		Least affluent	0.8 (0.5–1.2)	1.1 (0.5–2.3)	1.1 (0.5–2.2)	0.7 (0.5–0.9)
Women	0-1 health conditions	Most affluent	0.7 (0.5–1.0)	0.6 (0.3–1.6)	1.0 (0.5–1.9)	0.5 (0.3–0.8)
		Moderately affluent	0.7 (0.5–1.1)	0.8 (0.4–1.3)	3.5 (1.5–8.2)	0.7 (0.5–1.1)
		Least affluent	0.8 (0.5–1.2)	0.4 (0.1–1.1)	1.1 (0.6–2.4)	1.1 (0.7–1.6)
	2+ health conditions	Most affluent	0.6 (0.4–0.9)	0.8 (0.3–1.8)	0.7 (0.4–1.3)	1.1 (0.9–1.3)
		Moderately affluent	0.6 (0.4–0.8)	0.4 (0.1–1.3)	1.1 (0.6–2.0)	1.0 (0.8–1.2)
		Least affluent	1.0 (0.7–1.4)	0.5 (0.2–1.8)	1.2 (0.7–2.0)	0.8 (0.7–1.0)

## 4 Discussion

Widening inequalities in LE by social deprivation have been reported [5]. Although the contribution of MLTCs to shorter life and disability-free life expectancy has been reported, no temporal comparisons have been made. Here we used two longitudinal population-based studies of people aged 65 years and over in England to consider the contribution of MLTCs to the previously reported widening inequalities in LE and DFLE by socioeconomic deprivation between 1991 and 2011 of 1.7 years for men and 2.4 years for women [4]. We show that these widening DFLE inequalities between the most and least affluent men and women were not due solely to the increasing prevalence of MLTCs in the least affluent, since DFLE inequalities increased in those without MLTCs (to 1.8 years for men and 2.5 years for women). Widening DFLE inequalities were also evident in those initially disability-free, suggesting that higher disability prevalence in the least affluent was also not a major contributor. Moreover, there was little evidence of an increased incidence of MLTCs in the least affluent men and women, compared to the most affluent, between 1991 and 2011, which may also have explained the widening DFLE inequalities by social deprivation. There was, however, some evidence for a reduction in the disabling consequences of MLTCs in 2011 compared to 1991 in the most affluent, with small reductions in the proportion of remaining life with disability, in contrast to an increase for the least affluent. We conclude therefore that, whilst MLTCs are potentially part of the reason for widening inequalities in DFLE, they are not the major contributor, since DFLE inequalities of a similar magnitude are evident in older men and women without MLTCs.

In contrast to other studies, we also explored which underlying transitions in DFLE contributed to the widening inequalities between the most and least affluent older people. We found sex differences in those with and without MLTCs as we had found overall, [4] with the most affluent men seeing a lower risk of death from a disability-free state in 2011 than 1991 but the least affluent seeing a lower risk of death from disability (resulting in an increase in years with disability). The most affluent women in contrast saw reductions in the incidence of disability between 1991 and 2011, irrespective of MLTCs.

CFAS I and CFAS II are large studies, representative of the UK population aged 65 years or over. They offer a rare opportunity for estimating accurate time trends as the areas in which they were conducted, their sampling strategy, approach of participants and recruitment were identical. Both studies included people living in care settings and assisted living facilities, important since the percentage of people with MLTCs has grown more in these places of residence than for those living in the community [20]. We included more health conditions in the MLTCs measure than in other studies, making it less likely the comparison group without MLTCs had health conditions that were not recorded. There were, however, some limitations related to ascertainment and analysis of MLTCs, the measure of socioeconomic status, the CFAS I and II response rates and number of follow-up interviews, and validation of the estimates of LE and DFLE from IMaCh. Firstly, the majority of the health conditions were self-reported, albeit of a previous doctor diagnosis, rather than from medical records, although vision and hearing problems also included interviewer observation. Although self-report of health conditions is dependent on the participant's memory, information from informant interviews conducted with a friend or family member could replace information on missing health conditions (and other variables). Although medical records may not be biased by the participant's memory, other biases can occur, for instance lower attendance by those in lower socioeconomic deprivation groups. We acknowledge that the MLTCs measure was simplistic as a binary measure (0-1 versus 2 or more health conditions), instead of considering either clusters of diseases or MLTCs as a count. However, as LE and DFLE estimates were stratified by deprivation as well as MLTCs this choice was necessary due to the number of transitions in each group, as more MLTCs categories would have resulted in too low numbers for convergence. Secondly, although we chose area deprivation as our measure of socioeconomic deprivation to be more indicative of a participant's current rather than previous status (such as education or occupation), it is an area level rather than individual level measure based on possessions or pension income. Thirdly, in terms of the samples themselves, the CFAS II participation rate for the baseline interview was lower than that for CFAS I. Nevertheless, factors associated with non-response remained similar [12] and inverse probability weights were used to ensure population representativeness at each time point. In addition, the studies were based almost entirely on areas with high white British populations and therefore cannot represent the British Black and Minority Ethnic (BAME) community, for which dedicated studies are required to address this knowledge gap. Race could not be included in the inverse probability weights as we do not have this information for people who did not participate in the studies. CFAS I has further follow-up interviews after two years, however, CFAS II only has a follow-up interview at two. To replicate the analysis in CFAS I and II, follow up had to be restricted in CFAS I to only two years of interviews with 4.5 years of follow-up for vital status. Finally, although comparison of the estimates of LE at age 65 and 85 from our analysis using IMaCh were close to those published for England by the Office for National Statistics, we could not validate our estimates of DFLE as the Office for National Statistics use cross-sectional methods and a different measure of disability. Furthermore, we could not compare our estimates from discrete time models with those from continuous time models since the software for the latter [21] cannot accommodate weights which were necessary due to the design of CFAS.

We broaden the scope of previous literature by reporting the differential impact of MLTCs on inequalities in DFLE by socioeconomic deprivation. Although we did not look at the addition of each health condition individually, when comparing those with MLTCs to those without, we found that both LE and DFLE are reduced for those with MLTCs, similar to previous research [8,9,11], and to a similar degree across all deprivation groups. The latter is in contrast to differences by race [11] where MLTCs reduced LE more for white Americans than African Americans, and yet the resulting increase in percentage of life spent with disability was greater for African Americans than for white Americans, particularly for men. However, small group numbers may mean that differences between groups are imprecise.

Our study is the first to our knowledge to investigate the contribution of MLTCs to trends in LE and DFLE between deprivation groups. We found that, although the prevalence of MLTCs only increased for the least affluent, this was not the sole reason for widening DFLE inequalities by social deprivation, since widening DFLE inequalities were also present in those without MLTCs. Inequalities in LE and DFLE in those without MLTCs could be due to the causes of the causes such as physical activity, smoking, alcohol consumption and diet. As well as these behavioural factors contributing to the development of MLTCs [6,22,23], they have direct associations with disability [24]. However, there is disagreement on whether behavioural factors equally reduce LE [6,23], DFLE [23], and healthy life expectancy [6] across all socioeconomic groups or differentially across socioeconomic groups [22,25]. Between the generations included in CFAS I and CFAS II there were some improvements to early life factors, such as increases in the compulsory school leaving age in England. However, baseline CFAS II interviews began in 2008 so the full impact of austerity measures on health, which unequally impacted the more vulnerable [26,27], would not be measured between these two time points. With this in mind, to prevent inequalities widening further, a life-course perspective on the wider social determinants of health has been suggested [1,28,29]. Recommendations include restoring secondary school funding per pupil to previous levels, creating fair employment opportunities through increasing the national living wage and supporting in work training [1]. Built environment is associated with cognitive impairment [30,31] and disability [32,33]. In order to also ensure safe and healthy living environments, investment needs to be focussed on more deprived communities [1]. Providing access to more natural environments [31] and better infrastructure [34] could result in improvements to health and wellbeing.

Recently, the UK Government stated its aim to increase healthy life expectancy by five years before 2035 while also reducing inequalities [35]. The realisation of this aim may be substantially delayed since COVID-19 has disproportionately affected the health and mortality of the least affluent [36], in part thought to be due to the difference in prevalence of comorbid health conditions [37,38]. Individuals with MLTCs have complex health care needs. In health care systems based on specialisation in the treatment and care of people with single health conditions, as in the case of primary care in the UK, this will disproportionately impact more deprived groups where prevalence of MLTCs is rising. Providing support for health care professionals, especially in primary care where time is limited, both in terms of systems which encourage a multiple, rather than single, conditions management approach [39], and also access to additional clinical expertise and treatments to reduce unnecessary polypharmacy and increase patient safety, is urgently needed [22,40,41]. Social prescribing, where a link worker based in primary care supports people with MLTCs to co-produce a personalised social prescription to address behavioural change, is a promising intervention to improve wellbeing and low mood [42], especially in more deprived communities [43]. Social prescribing may be a welcome change to taking multiple prescription drugs for different health conditions. For maximum impact both downstream (within health care systems) and upstream solutions, such as unpolluted outdoor spaces, greater well-paid employment opportunities with safe working conditions and access to high quality neighbourhood services such as housing, public transport and schools, should be considered to delay the onset of long-term conditions and reduce MLTCs in more deprived communities.

## Contributors

CJ conceived the project, gained the funding (PI on project and co-applicant of CFAS), verified the data and co-wrote the paper. HB verified underlying data, conducted the analyses and wrote the first draft of the paper. FEM is deputy director and co-applicant of CFAS and project, verified underlying data, supervised the analysis, and commented on drafts. AK is co-applicant on project, supervised the analysis and commented on drafts. IL led the systematic review (to which CJ, HB and AK contributed), and commented on drafts. LR, LC and CB contributed to interpretation and commented on drafts. CB is the principal investigator for CFAS.

## Data sharing statement

The full study protocol can be found at http://www.cfas.ac.uk/cfas-ii/cfasii-study-design/. All CFAS I and CFAS II de-identified data is freely available upon application after approval from the CFAS management committee. The application form and essential data information can be found at http://www.cfas.ac.uk/cfas-ii/cfasii-data/. All interview questionnaires are provided at http://www.cfas.ac.uk/cfas-ii/cfasii-documents/.

## Funding

This work was supported by the Dunhill Medical Trust. CFAS II was supported by the UK Medical Research Council (MRC; research grant G0601022), Alzheimer's Society Grant Ref: 294, and received support from the UK National Institute for Health Research (NIHR) comprehensive clinical research networks in West Anglia and Trent, and the Dementias and Neurodegenerative Disease Research Network in Newcastle. HB is supported by the Dunhill Medical Trust (grant number RPGF1806\44), and AK by a Newcastle University Research Fellowship. This research was undertaken within the UK NIHR collaboration for leadership in applied health research and care for Cambridgeshire and Peterborough and the Cambridge Biomedical Research Centre infrastructures, Nottingham city and Nottinghamshire county NHS primary care trusts, and UK NIHR Policy Research Programme, conducted through the NIHR Older People and Frailty Policy Research Unit, PR-PRU-1217-21502.

## Declaration of Competing Interest

Dr. Bennett reports grants from Dunhill Medical Trust, grants from Medical Research Council, grants from Alzheimer's Society, grants from National Institute of Health Research, during the conduct of the study;

Dr. Kingston reports grants from Dunhill Medical Trust, grants from Legal & General, from UKRI Innovate UK, from NIHR School for Primary Care Research, during the conduct of the study; .Dr. Kingston reports grants from Dunhill Medical Trust, grants from Legal & General, grants from UKRI Innovate UK, grants from NIHR School for Primary Care Research, during the conduct of the study;Dr. Lourida reports grants from Dunhill Medical Trust, grants from Medical Research Council, grants from National Institute of Health Research, grants from Alzheimer's Society, during the conduct of the study;

Dr. Robinson reports grants from Dunhill Medical Trust, grants from Medical Research Council, grants from Alzheimer's Society, grants from National Institute of Health Research, during the conduct of the study;

Dr. Corner reports grants from Dunhill Medical Trust, grants from Medical Research Council, grants from Alzheimer's Society, grants from National Institute of Health Research, during the conduct of the study;

Dr. Brayne reports grants from Dunhill Medical Trust, grants from Medical Research Council, grants from Alzheimer's Society, grants from National Institute of Health Research, during the conduct of the study;

Dr. Matthews reports grants from Dunhill Trust, NIHR, Medical Research Council, and the Alzheimer's Society UK, during the conduct of the study;

Dr. Jagger reports grants from Dunhill Medical Trust, grants from Medical Research Council, grants from Alzheimer's Society, grants from National Institute of Health Research, during the conduct of the study.

## Appendix Supplementary materials

Image, application 1

## References

[1] Marmot M, Allen J, Boyce T, Goldblatt P, Morrison J. (2020). Health equity in England: the Marmot Review 10 years on.

[2] ONS. Health state life expectancies by national deprivation deciles, England and Wales: 2016 to 2018. ONS; 2020. Available from: https://www.gov.uk/government/statistics/health-state-life-expectancies-by-national-deprivation-deciles-england-and-wales-2016-to-2018.

[3] Aburto JM, Kashyap R, Schöley J (2021). Estimating the burden of the COVID-19 pandemic on mortality, life expectancy and lifespan inequality in England and Wales: a population-level analysis. J Epidemiol Commun Health.

[4] Bennett HQ, Kingston A, Spiers G (2021). Healthy ageing for all? Comparisons of socioeconomic inequalities in health expectancies over two decades in the Cognitive Function and Ageing Studies I and II. Int J Epidemiol.

[5] Bennett JE, Pearson-Stuttard J, Kontis V, Capewell S, Wolfe I, Ezzati M. (2018). Contributions of diseases and injuries to widening life expectancy inequalities in England from 2001 to 2016: a population-based analysis of vital registration data. The Lancet Public Health.

[6] Chan MS, van den Hout A, Pujades-Rodriguez M (2019). Socio-economic inequalities in life expectancy of older adults with and without multimorbidity: a record linkage study of 1.1 million people in England. Int J Epidemiol.

[7] Chireh B, D'Arcy C (2020). Contrasting trends in prevalence of chronic diseases and multimorbidity, Canada 1978–2014. SN Compr Clin Med.

[8] Reynolds SL, Haley WE, Kozlenko N. (2008). The impact of depressive symptoms and chronic diseases on active life expectancy in older Americans. Am J Geriatr Psychiatry.

[9] Reynolds SL, McIlvane JM. (2009). The impact of obesity and arthritis on active life expectancy in older Americans. Obesity.

[10] Jagger C, Matthews R, Matthews F, Robinson T, Robine JM, Brayne C. (2007). The burden of diseases on disability-free life expectancy in later life. J Gerontol A Biol Sci Med Sci.

[11] Laditka JN, Laditka SB. (2016). Associations of multiple chronic health conditions with active life expectancy in the United States. Disabil Rehabil.

[12] Gao L, Green E, Barnes LE (2015). Changing non-participation in epidemiological studies of older people: evidence from the cognitive function and ageing Study I and II. Age Ageing.

[13] Matthews FE, Arthur A, Barnes LE (2013). A two-decade comparison of prevalence of dementia in individuals aged 65 years and older from three geographical areas of England: results of the Cognitive Function and Ageing Study I and II. Lancet North Am Ed.

[14] Cognitive Function and Ageing Studies. CFAS II Study Design. 2021. http://www.cfas.ac.uk/cfas-ii/cfasii-study-design/ (Accessed 17 May 2021).

[15] Spiers N, Matthews R, Jagger C (2005). Diseases and impairments as risk factors for onset of disability in the older population in England and Wales: findings from the Medical Research Council cognitive function and ageing study. J Gerontol.

[16] Townsend P. (1979). Poverty in the United Kingdom.

[17] Townsend P. (1988). Health and Deprivation: Inequality and the North.

[18] Folstein MF, Folstein SE, McHugh PR. (1975). Mini-Mental State" A Practical method for grading the cognitive state of patients for the clinician. J Psychiatry Res.

[19] Lièvre A, Brouard N, Heathcote C. (2003). The estimation of health expectancies from cross-longitudinal surveys. Math Popul Stud.

[20] Matthews FE, Bennett H, Wittenberg R, Jagger C, Dening T, Brayne C. (2016). Who lives where and does it matter? Changes in the health profiles of older people living in long term care and the community over two decades in a high income country. PLoS One.

[21] Jackson C. (2011). Multi-State Models for Panel Data: The msm Package for R. J Stat Softw.

[22] Academy of Medical Sciences (2018). Multimorbidity: a priority for global health research. AMS.

[23] Kingston A, Byles J, Kiely K, Anstey K, Jagger C. (2020). The impact of smoking and obesity on disability-free life expectancy in older Australians. J Gerontol A Biol Sci Med Sci.

[24] Singer L, Green M, Rowe F, Ben-Shlomo Y, Morrissey K. (2019). Social determinants of multimorbidity and multiple functional limitations among the ageing population of England, 2002-2015. SSM Popul Health.

[25] Maki NE, Martikainen PT, Eikemo T (2014). The potential for reducing differences in life expectancy between educational groups in five European countries: the effects of obesity, physical inactivity and smoking. J Epidemiol Commun Health.

[26] British Medical Association (2016). Health in all policies: health, austerity and welfare reform.

[27] Stuckler D, Reeves A, Loopstra R, Karanikolos M, McKee M. (2017). Austerity and health: the impact in the UK and Europe. Eur J Public Health.

[28] Marengoni A, Calderon-Larrañaga A. (2020). Health inequalities in ageing: towards a multidimensional lifecourse approach. The Lancet Public Health.

[29] Wu Y-T, Daskalopoulou C, Muniz Terrera G (2020). Education and wealth inequalities in healthy ageing in eight harmonised cohorts in the ATHLOS consortium: a population-based study. The Lancet Public Health.

[30] Wu YT, Prina AM, Brayne C. (2015). The association between community environment and cognitive function: a systematic review. Soc Psychiatry Psychiatr Epidemiol.

[31] Wu YT, Prina AM, Jones A, Matthews FE, Brayne C. (2017). The built environment and cognitive disorders: results from the cognitive function and Ageing Study II. Am J Prev Med.

[32] Clarke P, Ailshire JA, Bader M, Morenoff JD, House JS. (2008). Mobility disability and the urban built environment. Am J Epidemiol.

[33] Rosso AL, Auchincloss AH, Michael YL. (2011). The urban built environment and mobility in older adults: a comprehensive review. J Aging Res.

[34] Williams DR, Costa MV, Odunlami AO, Mohammed SA. (2008). Moving upstream: How interventions that address the social determinants of health can improve health and reduce disparities. J Public Health Manag Pract.

[35] Department of Health and Social Care. Prevention is better than cure: our vision to help you live well for longer. DHSC, 2018. Available from: https://www.gov.uk/government/publications/prevention-is-better-than-cure-our-vision-to-help-you-live-well-for-longer.

[36] Office for National Statistics. Deaths involving covid-19 by local area and socioeconomic deprivation: deaths occurring between 1 March and 31 July 2020. 28 August 2020. Available from: https://www.ons.gov.uk/peoplepopulationandcommunity/birthsdeathsandmarriages/deaths/bulletins/deathsinvolvingcovid19bylocalareasanddeprivation/deathsoccurringbetween1marchand31july2020.

[37] Bambra C, Riordan R, Ford J, Matthews F. (2020). The COVID-19 pandemic and health inequalities. J Epidemiol Community Health.

[38] The Lancet Public H (2021). COVID-19 — break the cycle of inequality. The Lancet Public Health.

[39] Thorn J, Man M-S, Chaplin K (2020). Cost-effectiveness of a patient-centred approach to managing multimorbidity in primary care: a pragmatic cluster randomised controlled trial. BMJ Open.

[40] Barnett K, Mercer SW, Norbury M, Watt G, Wyke S, Guthrie B. (2012). Epidemiology of multimorbidity and implications for health care, research, and medical education: a cross-sectional study. Lancet North Am Ed.

[41] Davies LE, Spiers G, Kingston A, Todd A, Adamson J, Hanratty B. (2020). Adverse outcomes of polypharmacy in older people: systematic review of reviews. J Am Med Dir Assoc.

[42] Chatterjee HJ, Camic PM, Lockyer B, Thomson LJM. (2017). Non-clinical community interventions: a systematised review of social prescribing schemes. Arts Health.

[43] Tierney S, Wong G, Roberts N (2020). Supporting social prescribing in primary care by linking people to local assets: a realist review. BMC Med.

