# Supplementary material for: The contribution of multiple long-term conditions to widening inequalities in disability-free life expectancy over two decades: Longitudinal analysis of two cohorts using the Cognitive Function and Ageing Studies
Source: eClinicalMedicine. 2021 Jul 31;39:101041. doi: 10.1016/j.eclinm.2021.101041 (PMC8342913; doi:10.1016/j.eclinm.2021.101041)
Supplement: Supplementary file 1 [file mmc1.docx]

**Appendix**

*Statistical methods*

Markov chains describe transitions between different states over time where time is modelled discretely or continuously and the transitions between states are defined by transition probabilities. Here, a three state model was estimated using interpolated Markov chain modelling, in which time was modelled discretely. There were two non-absorbing states, disability-free (state 1) and with disability (state 2) and one absorbing state, death (state 3). Methods described here are from Lievre et al.[1]

If $X(x)$ denotes the state of an individual aged $x$ and $X(x+m)$ is their state after time $m$ has elapsed, then the transition probability is given by

$${}_{m}{p_{x}^{ij}=\Pr\left( X\left( x+m \right)=j \right|X\left( x \right)=i)}$$

where ${{}_{m}p}_{x}^{ij}$ is the entry $(i, j)$ in the transition probability matrix

$${}_{m}{P_{x}}=\left( \begin{matrix} {{}_{m}p}_{x}^{11} & {}_{m}{p_{x}^{12}} & {}_{m}{p_{x}^{13}} \\ {}_{m}{p_{x}^{21}} & {}_{m}{p_{x}^{22}} & {}_{m}{p_{x}^{23}} \\ 0 & 0 & 1 \end{matrix} \right) .$$

The transition probability ${{}_{m}p}_{x}^{ij}$ can be parameterised through the multinomial logistic regression model

$$ln\left( \frac{{}_{m}{p_{x}^{ij}}}{{}_{m}{p_{x}^{ii}}} \right)= \alpha_{ij}\left( m \right)+ \beta_{ij}\left( m \right)x , i\neq j (1)$$

where $m$ is a fixed time step of $m= 1 month$ and ${}_{m}{p_{x}^{ii}}$ is the probability of remaining in state $i$ after time $m$ has elapsed.

Given that the gap between baseline and follow-up interview in the Cognitive Function and Ageing Studies (CFAS I and CFAS II) was two years, the probability of transitioning within those two years can be found from the product of transition probability matrices:

$$\prod_{u=1}^{n} {}_{m}{P_{x+\left( u-1 \right)m}}$$

where $n$ is the number of steps $m$ between interviews (in this case $n=24$). The same product of matrices can be used for vitals follow-up between interview and death, however $n$ would vary as exact date of death was known. Therefore, for any given time interval $\left( x, x+y \right)$ where $y=nm$, the transition probability ${}_{y}{p_{x}^{ij}}$ is the $(i, j)$ entry of the matrix product $\left( {}_{m}{P_{x})({}_{m}{P_{x+m}}} \right)({}_{m}{P_{x+2m})}\ldots({}_{m}{P_{y-m}})$. The individual’s contribution to the likelihood is then the product of transition probability matrices over the study period and the likelihood can be maximised to estimate the parameters $\alpha_{ij}\left( m \right)$ and $\beta_{ij}\left( m \right)$ from equation $(1)$.

The transition probabilities can directly be used to estimate health expectancies. The health expectancy $e_{x}^{ij}$, the expected time spent in state $j$ by an individual who was in state $i$ at age $x$ is given by

$$e_{x}^{ij}= \sum_{y=1}^{\infty} {}_{y}{p_{x}^{ij}} .$$

The probabilities ${}_{y}{p_{x}^{ij}}(\theta)$ are estimated by ${}_{y}{p_{x}^{ij}}(\hat{\boldsymbol{\theta}})$ where $\hat{\boldsymbol{\theta}}$ is the vector of maximum likelihood estimates for the parameters of equation $(1)$, such that the health expectancies can be estimated from

$$\hat{e_{x}^{ij}}= e_{x}^{ij}(\hat{\boldsymbol{\theta}}) = \sum_{y=1}^{\infty} {}_{y}{p_{x}^{ij}}(\hat{\boldsymbol{\theta}}) .$$

Weighted averages of $e_{x}^{1j}$ and $e_{x}^{2j}$ give the overall health expectancy of state $j$ regardless of the starting state and the weights are the proportion of the sample in state 1 ($\pi_{x}^{1}$) and state 2 ($\pi_{x}^{2}$) at age $x$, such that

$$e_{x}^{.j}= \pi_{x}^{1}e_{x}^{1j}+\pi_{x}^{2}e_{x}^{2j} .$$

As an example, disability-free life expectancy at age 65 would be given by

$$e_{65}^{.1}= \pi_{65}^{1}e_{65}^{11}+\pi_{65}^{2}e_{65}^{21} .$$

**Appendix Table 1**: Comparison of estimates of life expectancy (LE) at ages 65 and 85 from the Cognitive Function and Ageing Studies (CFAS) IMaCh models to period life tables for England from the Office for National Statistics (ONS). Comparison to 1991-93 ONS life expectancy for CFAS I men and women and comparison to 2009-2011 ONS life expectancy for CFAS II men and women.

|  |  | **IMaCh** | **ONS England** |
| --- | --- | --- | --- |
|  |  | **Years** | **Years** |
| *LE at age 65* |  |  |  |
| CFAS I men |  | 14.1 | 14.3 |
| CFAS I women |  | 17.8 | 18.0 |
| CFAS II men |  | 17.7 | 18.2 |
| CFAS II women |  | 20.2 | 20.8 |
| *LE at age 85* |  |  |  |
| CFAS I men |  | 4.7 | 4.7 |
| CFAS I women |  | 6.0 | 5.9 |
| CFAS II men |  | 5.7 | 5.8 |
| CFAS II women |  | 6.6 | 6.9 |

**Appendix references**

1. Lièvre, A., N. Brouard, and C. Heathcote, *The Estimation of Health Expectancies from Cross-Longitudinal Surveys.* Mathematical Population Studies, 2003. **10**(4): p. 211-248.
